# 3-[2-(3-Methyl­quinoxalin-2-yl­oxy)eth­yl]-1,3-oxazolidin-2-one

**DOI:** 10.1107/S1600536810012687

**Published:** 2010-04-10

**Authors:** Caleb Anothane Ahoya, Rachid Bouhfid, Ballo Daouda, El Mokhtar Essassi, Lahcen El Ammari

**Affiliations:** a Laboratoire de Chimie Organique Hétérocyclique, Pôle de Compétences, Pharmacochimie, Avenue Ibn Battouta, BP 1014, Faculté des Sciences, Université Mohammed V-Agdal, Rabat, Morocco; b Institute of Nanomaterial and Nanotechnology, Avenue Armée Royale, Rabat, Morocco; c Laboratoire de Chimie du Solide Appliquée, Faculté des Sciences, Université Mohammed V-Agdal, Avenue Ibn Battouta, BP 1014, Rabat, Morocco

## Abstract

Two isomers were isolated during the reaction between 3-methyl­quinoxalin-2-one and bis­(2-chloro­ethyl)amine hydro­chloride. The crystal structure of one isomer has already been reported [Caleb, Bouhfid, Essassi & El Ammari (2009). *Acta Cryst.* E**65**, o2024–o2025], while that of the second isomer is the subject of this work. The title compound, C_14_H_15_N_3_O_3_, has a new structure containing oxazolidine and quinoxaline rings linked by an eth­oxy group. The main difference between the two isomers is the position of the oxazolidine group with respect to the quinoxaline system. The dihedral angle between the fused planar rings and the oxazolidin-2-one ring is 41.63 (8)° in the title mol­ecule.

## Related literature

For the biological activity of 3-[2-(3-methyl-1,2-dihydro­quin­oxalin-2-yl­oxy)eth­yl]oxazolidin-2-one, see: Madhusudhan *et al.* (2004 ▶); Soad *et al.* (2006 ▶); Sriharsha & Shashikanth (2006 ▶); Menoret *et al.* (2009 ▶); Wilhelmsson *et al.* (2008 ▶). For the structure of the isomer of the title compound, see: Caleb *et al.* (2009 ▶). For related structures, see: Doubia *et al.* (2007 ▶); Mamedov *et al.* (2007 ▶); Aschwanden *et al.*(1976 ▶)
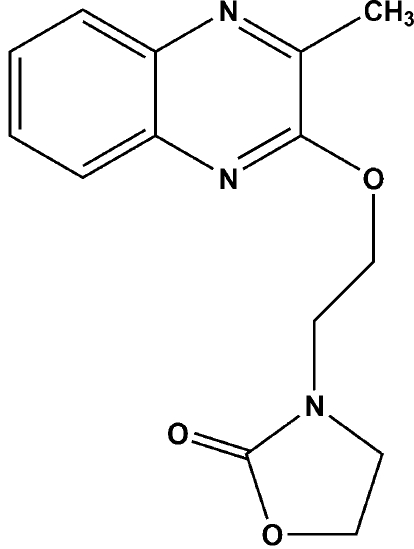


## Experimental

### 

#### Crystal data

C_14_H_15_N_3_O_3_
*M* *_r_* = 273.29
Triclinic, 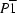

*a* = 6.9936 (3) Å
*b* = 7.6916 (3) Å
*c* = 13.3709 (6) Å
α = 86.649 (2)°
β = 77.044 (2)°
γ = 71.141 (2)°
*V* = 663.23 (5) Å^3^
*Z* = 2
Mo *K*α radiation
μ = 0.10 mm^−1^
*T* = 296 K
0.41 × 0.33 × 0.20 mm

#### Data collection

Bruker X8 APEXII CCD area-detector diffractometer
15358 measured reflections
3030 independent reflections
2358 reflections with *I* > 2σ(*I*)
*R*_int_ = 0.023

#### Refinement

*R*[*F* ^2^ > 2σ(*F* ^2^)] = 0.040
*wR*(*F* ^2^) = 0.121
*S* = 1.06
3030 reflections
198 parameters
H atoms treated by a mixture of independent and constrained refinement
Δρ_max_ = 0.21 e Å^−3^
Δρ_min_ = −0.17 e Å^−3^

### 

Data collection: *APEX2* (Bruker, 2005 ▶); cell refinement: *APEX2*; data reduction: *APEX2*; program(s) used to solve structure: *SHELXS97* (Sheldrick, 2008 ▶); program(s) used to refine structure: *SHELXL97* (Sheldrick, 2008 ▶); molecular graphics: *ORTEP-3 for Windows* (Farrugia,1997 ▶) and *PLATON* (Spek, 2009 ▶); software used to prepare material for publication: *WinGX* (Farrugia, 1999 ▶).

## Supplementary Material

Crystal structure: contains datablocks global, I. DOI: 10.1107/S1600536810012687/dn2552sup1.cif

Structure factors: contains datablocks I. DOI: 10.1107/S1600536810012687/dn2552Isup2.hkl

Additional supplementary materials:  crystallographic information; 3D view; checkCIF report

## Figures and Tables

**Fig. 1.**
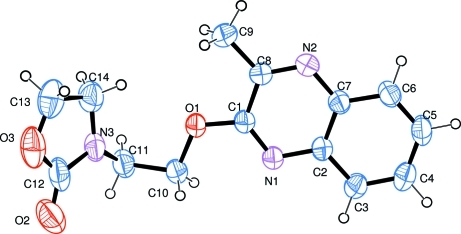
: Molecular structure of the title compound with the atom-labelling scheme. Displacement ellipsoids are drawn at the 50% probability level. H atoms are represented as small circles.

**Fig. 2.**
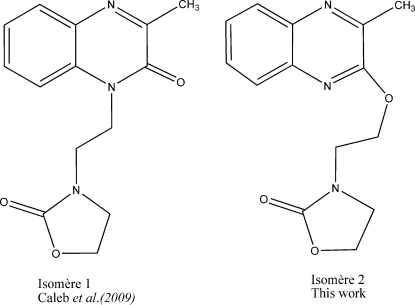
The structures of the two isomers.

## supplementary crystallographic information

### Comment

Oxazolidin-2-ones and quinoxalines are subjets of numerous articles in scientific journals concerning the development of new molecules as drug candidates such as antibacterials (Madhusudhan *et al.* 2004); (Sriharsha & Shashikanth, 2006), anti-viral (Wilhelmsson, *et al.* 2008), anti-tumor (Soad *et al.*2006),and anti-inflammatory (Menoret *et al.* 2009). Our investigation is intended to increase the biological activity of such molecules. During the synthesis, two isomers were isolated, and the structure of isomer 1 has already been published (Caleb *et al.* 2009) while that of ismer 2 is the subject of the present work.

The structure of the 3-(2-(3-methyl-1,2-dihydro-quinoxalin-2-yloxy)ethoxy) oxazolidin-2-one molecule is also built up from two fused six-membered rings linked to a five-membered ring (oxazolidin-2-one) by an ethoxy group, as shown in Fig.1. It would be interesting to compare the crystal structures of both isomers of this compound (scheme 1). Actually, the geometric parameters (bond lenghths and angles) of the two isomers are very similar to those observed in other heterocyclic structures (Aschwanden *et al.*, 1976; Doubia *et al.*, 2007; Mamedov *et al.*, 2007). However, the main difference between the two isomers is the position of the oxazolidine group with respect to the quinoxalin. Moreover, the dihedral angle between the fused six-membered rings and the five cycles measuring 20.04 (9)° in the isomer 1 instead of 41.63 (8)° in the isomer 2.

### Experimental

In a 100 ml flask, is reacted 0.0125 moles of quinoxalin-2-one with 2.66 moles of dichloroethylamine hydrochloride in 40 ml of dimethyl formamide in presence of 2.87 moles of potassium carbonate and a few milligrams of tetran-butyl ammonium bromide. The mixture was brought to reflux in a sand bath, magnetic stirring and the reaction progress was monitored by thin layer chromatography. After evaporation of solvent under reduced pressure, the residue obtained is chromatographed on silica column (hexane / acetate: 4 / 6). Thus we have isolated two compounds. Recrystallization occurred in the same eluent. This compound was obtained in 38% and his melting point is 169°C.

### Refinement

H atoms were located in a difference map and treated as riding with C—H = 0.96 Å for methyl groups and C—H = 0.93 Å for all other hydrogens with U_iso_(H) = 1.2 U_eq_(aromatic, methine ) or U_iso_(H) = 1.5 U_eq_(methyl). All other H atoms were located from difference Fourier maps and refined without any distance restraints.

### Crystal data

**Table:**

C_14_H_15_N_3_O_3_	*Z* = 2
*M**_r_* = 273.29	*F*(000) = 288
Triclinic, *P*1	*D*_x_ = 1.368 Mg m^−^^3^
Hall symbol: -P 1	Mo *K*α radiation, λ = 0.71073 Å
*a* = 6.9936 (3) Å	Cell parameters from 15358 reflections
*b* = 7.6916 (3) Å	θ = 2.8–27.5°
*c* = 13.3709 (6) Å	µ = 0.10 mm^−^^1^
α = 86.649 (2)°	*T* = 296 K
β = 77.044 (2)°	Prism, colourless
γ = 71.141 (2)°	0.41 × 0.33 × 0.20 mm
*V* = 663.23 (5) Å^3^	

### Data collection

**Table:**

Bruker X8 APEXII CCD area-detector diffractometer	2358 reflections with *I* > 2σ(*I*)
Radiation source: fine-focus sealed tube	*R*_int_ = 0.023
graphite	θ_max_ = 27.5°, θ_min_ = 2.8°
φ and ω scans	*h* = −9→9
15358 measured reflections	*k* = −9→9
3030 independent reflections	*l* = −17→17

### Refinement

**Table:**

Refinement on *F*^2^	Primary atom site location: structure-invariant direct methods
Least-squares matrix: full	Secondary atom site location: difference Fourier map
*R*[*F*^2^ > 2σ(*F*^2^)] = 0.040	Hydrogen site location: inferred from neighbouring sites
*wR*(*F*^2^) = 0.121	H atoms treated by a mixture of independent and constrained refinement
*S* = 1.06	*w* = 1/[σ^2^(*F*_o_^2^) + (0.0643*P*)^2^ + 0.0716*P*] where *P* = (*F*_o_^2^ + 2*F*_c_^2^)/3
3030 reflections	(Δ/σ)_max_ < 0.001
198 parameters	Δρ_max_ = 0.21 e Å^−^^3^
0 restraints	Δρ_min_ = −0.17 e Å^−^^3^

### Special details

**Table:**

Geometry. All esds (except the esd in the dihedral angle between two l.s. planes) are estimated using the full covariance matrix. The cell esds are taken into account individually in the estimation of esds in distances, angles and torsion angles; correlations between esds in cell parameters are only used when they are defined by crystal symmetry. An approximate (isotropic) treatment of cell esds is used for estimating esds involving l.s. planes.
Refinement. Refinement of *F*^2^ against ALL reflections. The weighted *R*-factor wR and goodness of fit *S* are based on *F*^2^, conventional *R*-factors *R* are based on *F*, with *F* set to zero for negative *F*^2^. The threshold expression of *F*^2^ > σ(*F*^2^) is used only for calculating *R*-factors(gt) etc. and is not relevant to the choice of reflections for refinement. *R*-factors based on *F*^2^ are statistically about twice as large as those based on *F*, and *R*- factors based on ALL data will be even larger.

### Fractional atomic coordinates and isotropic or equivalent isotropic displacement parameters (Å^2^)

**Table:**

	*x*	*y*	*z*	*U*_iso_*/*U*_eq_	
C1	0.31122 (17)	0.72867 (15)	0.95763 (8)	0.0378 (3)	
C2	0.08813 (17)	0.80823 (15)	1.11146 (8)	0.0382 (3)	
C3	−0.11007 (19)	0.83829 (18)	1.17492 (10)	0.0470 (3)	
C4	−0.1442 (2)	0.8837 (2)	1.27638 (10)	0.0531 (3)	
C5	0.0143 (2)	0.90272 (19)	1.31801 (10)	0.0540 (3)	
C6	0.2068 (2)	0.87655 (18)	1.25750 (10)	0.0503 (3)	
C7	0.24799 (18)	0.82807 (16)	1.15335 (9)	0.0403 (3)	
C8	0.47691 (17)	0.75240 (16)	0.99773 (9)	0.0412 (3)	
C9	0.68526 (19)	0.7217 (2)	0.92895 (11)	0.0546 (3)	
H9A	0.7371	0.5987	0.9015	0.082*	
H9B	0.7784	0.7393	0.9674	0.082*	
H9C	0.6743	0.8075	0.8737	0.082*	
C10	0.21267 (19)	0.63937 (19)	0.81444 (9)	0.0465 (3)	
H10A	0.1105	0.7551	0.8041	0.056*	
H10B	0.1429	0.5643	0.8593	0.056*	
C11	0.3226 (2)	0.54186 (19)	0.71328 (10)	0.0523 (3)	
H11A	0.4218	0.4258	0.7258	0.063*	
H11B	0.2221	0.5146	0.6826	0.063*	
C12	0.3726 (3)	0.7066 (2)	0.55269 (11)	0.0625 (4)	
C13	0.6852 (3)	0.7454 (3)	0.54185 (14)	0.0820 (5)	
H13A	0.6957	0.8634	0.5570	0.098*	
H13B	0.8151	0.6739	0.4986	0.098*	
C14	0.6363 (3)	0.6450 (3)	0.63974 (11)	0.0682 (4)	
H14A	0.7319	0.5209	0.6375	0.082*	
H14B	0.6394	0.7098	0.6991	0.082*	
N1	0.12438 (14)	0.75658 (13)	1.01023 (7)	0.0406 (2)	
N2	0.44386 (15)	0.80020 (15)	1.09343 (8)	0.0462 (3)	
N3	0.43028 (18)	0.64507 (16)	0.64066 (8)	0.0534 (3)	
O1	0.36793 (12)	0.67107 (12)	0.85906 (6)	0.0460 (2)	
O2	0.2172 (2)	0.70974 (18)	0.52691 (9)	0.0879 (4)	
O3	0.5186 (2)	0.76983 (16)	0.49204 (8)	0.0829 (4)	
H3	−0.220 (2)	0.8300 (19)	1.1449 (11)	0.052 (4)*	
H4	−0.280 (2)	0.908 (2)	1.3175 (13)	0.068 (4)*	
H5	−0.009 (2)	0.934 (2)	1.3883 (14)	0.064 (4)*	
H6	0.322 (3)	0.889 (2)	1.2864 (13)	0.071 (4)*	

### Atomic displacement parameters (Å^2^)

**Table:**

	*U*^11^	*U*^22^	*U*^33^	*U*^12^	*U*^13^	*U*^23^
C1	0.0389 (6)	0.0433 (6)	0.0313 (6)	−0.0140 (5)	−0.0066 (4)	0.0011 (4)
C2	0.0413 (6)	0.0410 (6)	0.0317 (6)	−0.0135 (5)	−0.0066 (4)	0.0011 (4)
C3	0.0439 (7)	0.0559 (7)	0.0406 (7)	−0.0189 (6)	−0.0031 (5)	−0.0016 (5)
C4	0.0526 (7)	0.0592 (8)	0.0396 (7)	−0.0161 (6)	0.0039 (6)	−0.0019 (6)
C5	0.0656 (8)	0.0568 (8)	0.0317 (7)	−0.0106 (6)	−0.0064 (6)	−0.0048 (5)
C6	0.0545 (8)	0.0564 (8)	0.0388 (7)	−0.0113 (6)	−0.0162 (6)	−0.0043 (5)
C7	0.0415 (6)	0.0427 (6)	0.0354 (6)	−0.0106 (5)	−0.0097 (5)	−0.0003 (5)
C8	0.0366 (6)	0.0476 (6)	0.0391 (6)	−0.0127 (5)	−0.0083 (5)	−0.0005 (5)
C9	0.0394 (6)	0.0745 (9)	0.0505 (8)	−0.0210 (6)	−0.0050 (5)	−0.0066 (6)
C10	0.0446 (6)	0.0629 (8)	0.0351 (6)	−0.0211 (6)	−0.0079 (5)	−0.0038 (5)
C11	0.0608 (8)	0.0600 (8)	0.0381 (7)	−0.0217 (6)	−0.0093 (6)	−0.0066 (5)
C12	0.0842 (11)	0.0549 (8)	0.0358 (7)	−0.0028 (8)	−0.0139 (7)	−0.0097 (6)
C13	0.0972 (13)	0.0874 (12)	0.0545 (10)	−0.0363 (10)	0.0076 (9)	0.0021 (8)
C14	0.0723 (10)	0.0929 (11)	0.0440 (8)	−0.0373 (9)	−0.0061 (7)	0.0036 (7)
N1	0.0385 (5)	0.0515 (6)	0.0331 (5)	−0.0169 (4)	−0.0062 (4)	−0.0017 (4)
N2	0.0400 (5)	0.0571 (6)	0.0425 (6)	−0.0140 (5)	−0.0120 (4)	−0.0036 (5)
N3	0.0602 (7)	0.0644 (7)	0.0316 (5)	−0.0157 (5)	−0.0071 (5)	−0.0024 (5)
O1	0.0409 (4)	0.0667 (6)	0.0316 (4)	−0.0203 (4)	−0.0035 (3)	−0.0063 (4)
O2	0.1019 (9)	0.0914 (9)	0.0633 (8)	−0.0034 (7)	−0.0421 (7)	−0.0077 (6)
O3	0.1211 (10)	0.0797 (8)	0.0399 (6)	−0.0288 (7)	−0.0081 (6)	0.0096 (5)

### Geometric parameters (Å, °)

**Table:**

C1—N1	1.2932 (14)	C9—H9C	0.9600
C1—O1	1.3457 (13)	C10—O1	1.4398 (13)
C1—C8	1.4458 (15)	C10—C11	1.5041 (18)
C2—N1	1.3777 (14)	C10—H10A	0.9700
C2—C3	1.4083 (16)	C10—H10B	0.9700
C2—C7	1.4099 (15)	C11—N3	1.4523 (17)
C3—C4	1.3695 (18)	C11—H11A	0.9700
C3—H3	0.966 (14)	C11—H11B	0.9700
C4—C5	1.397 (2)	C12—O2	1.2046 (19)
C4—H4	0.949 (16)	C12—N3	1.3394 (19)
C5—C6	1.3646 (19)	C12—O3	1.357 (2)
C5—H5	0.948 (17)	C13—O3	1.424 (2)
C6—C7	1.4045 (17)	C13—C14	1.510 (2)
C6—H6	0.999 (17)	C13—H13A	0.9700
C7—N2	1.3793 (15)	C13—H13B	0.9700
C8—N2	1.3013 (15)	C14—N3	1.4379 (19)
C8—C9	1.4923 (16)	C14—H14A	0.9700
C9—H9A	0.9600	C14—H14B	0.9700
C9—H9B	0.9600		
			
N1—C1—O1	121.60 (10)	C11—C10—H10A	110.3
N1—C1—C8	124.34 (10)	O1—C10—H10B	110.3
O1—C1—C8	114.06 (9)	C11—C10—H10B	110.3
N1—C2—C3	119.79 (10)	H10A—C10—H10B	108.6
N1—C2—C7	120.95 (10)	N3—C11—C10	114.20 (11)
C3—C2—C7	119.25 (11)	N3—C11—H11A	108.7
C4—C3—C2	119.75 (12)	C10—C11—H11A	108.7
C4—C3—H3	121.5 (9)	N3—C11—H11B	108.7
C2—C3—H3	118.7 (8)	C10—C11—H11B	108.7
C3—C4—C5	121.01 (12)	H11A—C11—H11B	107.6
C3—C4—H4	118.6 (10)	O2—C12—N3	127.96 (16)
C5—C4—H4	120.3 (10)	O2—C12—O3	122.31 (14)
C6—C5—C4	120.14 (12)	N3—C12—O3	109.73 (14)
C6—C5—H5	118.9 (9)	O3—C13—C14	106.05 (14)
C4—C5—H5	120.9 (9)	O3—C13—H13A	110.5
C5—C6—C7	120.42 (12)	C14—C13—H13A	110.5
C5—C6—H6	120.9 (10)	O3—C13—H13B	110.5
C7—C6—H6	118.6 (10)	C14—C13—H13B	110.5
N2—C7—C6	119.74 (10)	H13A—C13—H13B	108.7
N2—C7—C2	120.85 (10)	N3—C14—C13	101.80 (14)
C6—C7—C2	119.41 (11)	N3—C14—H14A	111.4
N2—C8—C1	119.95 (10)	C13—C14—H14A	111.4
N2—C8—C9	120.35 (10)	N3—C14—H14B	111.4
C1—C8—C9	119.70 (11)	C13—C14—H14B	111.4
C8—C9—H9A	109.5	H14A—C14—H14B	109.3
C8—C9—H9B	109.5	C1—N1—C2	115.96 (9)
H9A—C9—H9B	109.5	C8—N2—C7	117.92 (10)
C8—C9—H9C	109.5	C12—N3—C14	112.13 (13)
H9A—C9—H9C	109.5	C12—N3—C11	122.09 (13)
H9B—C9—H9C	109.5	C14—N3—C11	123.42 (12)
O1—C10—C11	106.91 (10)	C1—O1—C10	117.34 (9)
O1—C10—H10A	110.3	C12—O3—C13	109.59 (12)
